# Supplementary material for: Postoperative management of total elbow arthroplasty: Results of a European survey among orthopedic surgeons
Source: PLoS One. 2022 Nov 14;17(11):e0277662. doi: 10.1371/journal.pone.0277662 (PMC9662720; doi:10.1371/journal.pone.0277662)
Supplement: S1 Appendix — (DOCX) [file pone.0277662.s001.docx]

**S1 APPENDIX — Survey: postoperative management of TEA**

1. What country are you from?
2. Sex
   1. Male
   2. Female
3. How many years of experience do you have performing TEAs?
4. How many TEAs do you perform annually?
5. How many different types of prosthesis do you use? Please specify the types.
6. Which implant do you (usually) use?
7. Which surgical approach do you (usually) use?
   1. Posterior approach, Triceps reflecting
   2. Posterior approach, Triceps-on
   3. Posterior approach, Triceps-flap
   4. Posterior approach, Triceps-split
8. Following TEA, do you use a (removable) cast?
   1. Yes
   2. No
9. How many days do you use a (removable) cast?
10. In which position is the elbow immobilized? (degrees of flexion).
11. Do you allow passive movement?
    1. Yes
    2. No
12. On what day would you start passive movement?
13. Do you allow active movement?
    1. Yes
    2. No
14. On what day would you start active movement?
15. Do you give specific lifelong recommendations on weight lifting?
    1. no limitation/recommendation
    2. <1kg lifelong
    3. 1-5kg lifelong
    4. 1-10kg lifelong
16. Do you give specific lifelong recommendations on axial loading?
    1. no limitation/recommendation
    2. <1kg lifelong
    3. 1-5kg lifelong
    4. 1-10kg lifelong
17. Do you give specific instructions on lifelong activities allowed?
    1. Yes
    2. No
18. What instructions do you give?
19. Do you give any other specific restrictions or instructions?
    1. Yes
    2. No
20. Which other restrictions or instructions?
21. Do you advise patients to be supervised by a physiotherapist postoperatively?
    1. Yes
    2. No
22. For how long do you advise them to be supervised by a physiotherapist?
23. When do you perform postoperative assessments?
24. How do you perform postoperative assessment?
25. Do you monitor triceps function?
    1. Yes
    2. No
26. How do you monitor it?
    1. Mrc scale
    2. Extension against resistance
    3. Active extension against gravity
    4. Clinical evaluation of strength
    5. Other…
27. Do you have different postoperative protocols for different indications?
    1. Yes
    2. No
28. Can you specify the differences?
29. Does your postoperative protocol depend on whether it is primary or revision surgery?
    1. Yes
    2. No
30. Can you specify the differences?
31. Do you wish to add any comments?
