# Supplementary material for: Postoperative management of total elbow arthroplasty: Results of a European survey among orthopedic surgeons
Source: PLoS One. 2022 Nov 14;17(11):e0277662. doi: 10.1371/journal.pone.0277662 (PMC9662720; doi:10.1371/journal.pone.0277662)
Supplement: Attachment — Submitted filename: Response to reviewers .docx [file pone.0277662.s003.docx]

PLOS one editorial office

Subject: response to Editor

August 21^th^ 2022

Dear Maria Elisabeth Johanna Zalm,

Please find enclosed our revised manuscript “Postoperative management of Total Elbow Arthroplasty: Results of a European survey among orthopedic surgeons”. Thanks for the comments of the reviewer and the opportunity to submit a revised version of current manuscript. As requested we have addressed and responded to the reviewer’s comments in order to improve the quality of the manuscript.

Yours sincerely,

On behalf of my co-authors,

Willemijn van Dam

Correspondence:

W. van Dam
Department of Orthopedics, University Medical Center Groningen, The Netherlands
Tel: +31-6-13701837
w.van.dam@student.rug.nl

**Reviewer 1:**

**Comment 1**
Reviewer #1: The 9% response rate seems low and the justification for why this was actually a good representation was confusing and could benefit from clarification.
**Response**
We agree this may have been confusing. We edited the manuscript in the abstract (line 32), the methods (line 71-72) and results section (line 104-107) to clarify the response rate.

**Comment 2**
The authors also mention the need for biomechanical studies in the conclusion but don't mention them throughout the paper so it might be beneficial to have a more fleshed out thought about that in the discussion and how that can relate to postop protocol standardizations since that seems to be the main focus.
**Response**
We added an extra statement (line 243-246) explaining that insight in the amount of axial loading and weight bearing allowed by the prosthesis will lead to more justified, standardized, postoperative advise.

**Comment 3**
Line 228 This is not really contradictory as immobilization may be for soft tissue rest. Many of these procedures are done in elderly patients with poor soft tissue envelopes or in the setting of geriatric trauma where the skin and subcutaneous tissue is very compromised.
**Response**
We agree with the reviewer. An advantage of the triceps sparing surgical approach is direct functional treatment of the elbow. However, it is in fact true that even with the triceps sparing surgical approach, postoperative immobilization may be advised due to soft tissue damage. We added a comment (line 225-227) about this in the manuscript.

**Comment 4**
Line 234 The authors need to explain that total elbow arthroplasty failure is not a complete mystery and finds its origin in nonanatomic force distribution, which leads to stress shielding and bone resorption over time. The violation of elbow biomechanics is outlined in an article in the Journal of Hand Surgery. Please add this reference so that the reader can understand why these fail quite reliably.
Kaufmann RA, D'Auria JL, Schneppendahl J. Total Elbow Arthroplasty: Elbow Biomechanics and Failure. J Hand Surg Am. 2019 Aug;44(8):687-692. doi: 10.1016/j.jhsa.2018.11.020. Epub 2019 Feb 13. PMID: 30770149.
**Response**
An explanation considering nonanatomic force distribution and its role in the failure of the prosthesis has been added (line 234-236) with reference to the suggested paper.
